# The Gut Microbiota-Host Partnership as a Potential Driver of Kawasaki Syndrome

**DOI:** 10.3389/fped.2019.00124

**Published:** 2019-04-05

**Authors:** Susanna Esposito, Ilaria Polinori, Donato Rigante

**Affiliations:** ^1^Pediatric Clinic, Department of Surgical and Biomedical Sciences, Università degli Studi di Perugia, Perugia, Italy; ^2^Institute of Pediatrics, IRCCS, Rome, Italy; ^3^Fondazione Policlinico Universitario A. Gemelli, IRCCS, Rome, Italy; ^4^Università Cattolica Sacro Cuore, Rome, Italy

**Keywords:** Kawasaki syndrome, infection, innovative biotechnologies, microbiota, personalized medicine, child

## Abstract

Kawasaki syndrome (KS) is a necrotizing vasculitis of small- and medium-sized vessels mostly affecting children under 5 years of age; a host of clinical and epidemiological data supports the notion that KS might result from an infectious disease. However, many efforts have failed to identify a potentially universal trigger of KS. The contribution of the intestinal microbial community—called the “microbiota”—to KS has been evaluated by an increasing number of studies, though limited to small cohorts of patients. Differences in the microbiota composition were found in children with KS, both its acute and non-acute phase, with abnormal colonization by *Streptococcus* species in the intestinal tract and a wider presence of Gram-positive cocci in jejunal biopsies. In particular, a higher number of Gram-positive cocci (of the genera *Streptococcus* and *Staphylococcus*), *Eubacterium, Peptostreptococcus*, and HSP60-producing Gram-negative microbes have been found in the stools of KS children, and their effects on the antigenic repertoire of specific T cells and Vβ2 T cell expansion have been assessed. Conversely, *Lactobacilli* were lacking in most children with KS compared with other febrile illnesses and healthy controls. All studies available to date have confirmed that an imbalance in the gut microbiota might indirectly interfere with the normal function of innate and adaptive immunity, and that variable microbiota interactions with environmental factors, mainly infectious agents, might selectively drive the development of KS in genetically susceptible children. Further investigations of the intestinal microflora in larger cohorts of KS patients will provide clues to disentangle the pathogenesis of this disease and probably indicate disease-modifying agents or more rational KS-specific therapies.

## Introduction

The most insidious primary vasculitis in childhood is Kawasaki syndrome (KS), an acute multi-systemic illness which predominantly affects children under 5 years of age (1). Currently, this disorder of unknown etiology remains the main cause of acquired heart disease among children living in developed countries, where rheumatic fever has been surpassed (2, 3). This condition was originally called “mucocutaneous lymph node syndrome” by Dr. Tomisaku Kawasaki, who was its discoverer, and was thought to be a benign children's disease; nowadays, the illness has been described worldwide in children of every ethnicity following the presence of fever persisting (at least) 5 days together with (at least) 4 of the 5 following signs: bilateral conjunctival injection, oropharyngeal inflammation, abnormalities of hands and feet, polymorphous exanthema, and non-purulent cervical lymphadenopathy, usually unilateral (4). Several years after the first description, fatalities occurred among children with KS younger than 2 years living in Japan, prompting clinicians to reconsider KS's long-term risks related to systemic and necrotizing effects on the vascular endothelium of small- and medium-sized arteries, which have been acknowledged in all guidelines related to the management of KS (5, 6). The most relevant sequelae of KS include variable degrees of damage within coronary arteries in combination with angina, myocardial infarction, ischemic cardiomyopathy, and sudden death; these complications should be preventable with a timely treatment of high-dose intravenous immunoglobulin (IVIG), which is the recommended therapeutic strategy in KS (7). Higher acute phase reactants and younger age at onset of KS are nodal points in determining, respectively, a failure in the response to IVIG and an increased occurrence of coronary artery abnormalities (8). The prediction of IVIG resistance is also crucial in KS patients, as recognizing these high-risk children should consent to start an intensified treatment protocol combined with IVIG to prevent coronary injuries (9).

KS incidence varies widely among different ethnic groups; for instance, in the United Kingdom it has stabilized and remains low at 2.8 per 100,000 population under the age of 20 years. However, general practitioners should be aware that the condition occurs throughout childhood and across the seasons, and—given the potential cardiovascular sequelae—KS should be considered in all children with persistent fever, even in older children and adolescents (10). KS is most prominently recognized in Japan, Korea, and Taiwan, reflecting increased genetic susceptibility among Asian populations. A recent study reported an incidence of ~240 per 100,000 children under 4 years of age in Japan (11). There is still much controversy about the etiology of KS, though epidemiologic and clinical data suggest that KS might originate from an abnormal response to undisclosed infectious diseases in genetically susceptible children (12). There is no agreement whether KS-related infectious agents are of viral, bacterial or fungal origin (13), and the underlying immune mechanisms behind KS have not been completely highlighted, remaining only partially known. The absence of a proven unambiguous cause of KS has induced the scientific community to pay attention to other environmental hypothetical triggers, and in particular the composition of the resident intestinal flora as a potential contributor to KS has been evaluated by different research groups.

The main aim of this review was to analyze the relationship between the microbial community, or the “microbiota,” and the overall impact of bacterial or viral infections in the potential development of KS. Scientific papers have been searched from the electronic databases of PubMed until January 2019; the retrieving words were “Kawasaki disease,” “Kawasaki syndrome,” “microbiota,” and “microbiome”; additional reports were identified and analyzed through the specific references cited in the retrieved papers. Only papers published in English and those showing evidence-based data were included in our evaluation.

## Evolution of the Microbiota in Children

The microbiota, a microbial community of trillions of microorganisms and at least 1,000 different bacterial species, some eukaryotic fungi and viruses, and which covers every surface of the human body, plays a contributory role in many infections, immune-mediated disorders, rheumatologic diseases, and disorders of the nervous system. The microbiome, on the other hand, is the collection of the whole genome sequences of those microorganisms, consisting of more than 5,000,000 genes (14, 15). In particular, the gut microbiota is strictly linked to the chronological age of each individual and modulates host physiology and metabolism through different mechanisms.

Each stage of human life is characterized by a specific intestinal microbial composition: the microbiota that initially colonizes the fetus' intestinal tube consists of aerobic organisms such as *Enterococcus* and *Streptococcus*, is then gradually replaced by anaerobes such as *Bifidobacterium* and *Lactobacillus* and finally reaches the adult composition dominated by *Bacteroides* and Firmicutes (16, 17). The fetal microbiota is prone to be conditioned by the type of delivery; the mother's vaginal flora is a relevant source of *Lactobacillus, Prevotella*, and *Bifidobacterium*. Conversely, a cesarean delivery delays contact with these species, producing a similar-to-skin flora, dominated by Staphylococci (18). The feeding regimens and food supplements also play a role in modifying the resident flora; a greater complexity is normally seen in infants fed with formula, rather than in breastfed babies who have an “adult-like” structured microbiota with a population rich with Bifidobacteria, Lactobacilli, and *Bacteroides* (18). The infant gut microbiota is variable in composition over time and highly changeable during the first year of life, being influenced by specific bacteria to which a baby happens to be exposed, as shown by the resemblance of infants' stool microbial community with mothers' milk and vaginal samples (19). Thereafter, the infant's intestinal tract progresses toward an extremely dense colonization, ending with a mixture of microbes that is broadly very similar to an adult's intestine. During adulthood, the gut microbiota becomes stable and this intestinal homeostasis remains in equilibrium with the host. Food habits influence the composition of the whole intestinal microbiota, as testified by the lower prevalence of *Bacteroides* in those suffering from malnutrition and by different microbiota changes occurring in children with diet-related diseases, such as allergies and obesity (19).

Arumugam et al. identified three distinct enterotypes, namely *Bacteroides, Prevotella*, and *Ruminococcus*, which reflect many individual alimentary profiles: *Bacteroides* correlates to a high-fat or high-protein regimen, whereas *Prevotella* is associated with higher consumption of fibers and simple sugars (20). More recent data have consented to unify *Bacteroides* and *Ruminococcus* enterotypes due to the large similarity between the two (21). It is well-established that early events of birth, environmental factors during infancy, sex hormones, diet, body weight, and use of antibiotics can undoubtedly differentiate the composition of the microbiota (22, 23). There are no ideal culture methods to give us a real overview of the complete intestinal flora, though molecular methods, e.g., DNA microarrays with comprehensive coverage of most bacterial taxa represented in the available database of small subunit ribosomal RNA gene sequences, should allow the characterization of most taxonomic groups of the intestinal bacteria (24, 25).

## Role of the Bacterial Flora in Childhood Diseases

The ancient symbiosis between the human gastrointestinal tract and its resident microbiota involves diverse reciprocal interactions between the microbiota itself and the host, with relevant consequences for human health and physiology. The quality of the microbial flora has an impact on the maintenance of health and also on prevention of diseases. Indeed, there are numerous roles carried out by the intestinal ecosystem, as the stimulation of angiogenesis, control of host fat storage and protection against other pathogens. In particular, the microbiota influences the formation and progress of regulation of both innate and adaptive immunity in close interaction with the intestinal mucosal immune system. The intestinal mucosa may be considered as an immunological niche as it hosts a complex immune-functional organ comprised by T cell subpopulations, neutrophils, macrophage-dendritic cells, enterocytes (that possess tight intercellular junctions) and their related anti- and pro-inflammatory cytokines as well as several other mediators of inflammation or antimicrobial peptides, defensins, and secretory immunoglobulin A (IgA) (26). The intestinal microbiota may also have direct or indirect effects on the natural course of viral infections, interacting with viral particles and leading to differences in either pathogenicity or anti-viral immune response through recruitment and activation of several T cell subpopulations (18). A large amount of data has also depicted the relevance of gut microbiota-immune system cross-talk in several diseases, and indeed an “imbalance” of the intestinal flora has been shown in patients with atopic diseases and various non-infectious diseases, including metabolic disorders, chronic inflammatory bowel disease (IBD), irritable bowel syndrome, pancreatic diseases, atherosclerosis, and rheumatoid arthritis (27, 28). Furthermore, the gut microbiota, interacting with pattern recognition receptors (PRRs), signaling receptors that can recognize molecular structures of pathogens and activate the cascade of innate immunity, plays a crucial role in maintaining the homeostasis of the innate immunity responses in the gut, and leaks in the intestinal mucosal barrier lead to the translocation of bacterial products into portal circulation which promotes systemic inflammation (29). In addition, the microbiota can maintain a segregation between intestinal mucosa and bacteria via PRRs, though pathogens might usurp innate signals to their advantage (30).

Several immunologic, metabolic and nutritional processes are normally controlled by the intestinal microbiota, and changes in the local microbial communities have been linked to chronic low-grade inflammation (31). Alexander et al. demonstrated that the microbiota has specific effects on adaptive immunity, such as the induction of regulatory effector CD4+ cells and production of cytokines and antimicrobial factors, influencing the individual response to various environmental stimuli, as seen in IBD, Crohn's disease and ulcerative colitis (32). In a recent work, Schwiertz et al. have shown microbiota changes characterized by decreased numbers of *Faecalibacterium praunsitzii* and increased numbers of *Escherichia coli* in IBD (33). Additionally, in celiac disease De Palma et al. have described a difference in the microbiota composition with a reduction of *Bifidobacterium, Clostridium histolyticum, Clostridium lituseburense, Faecalibacterium prausnitzii*, and an abundance of *Bacteroides* and *Prevotella* strains (34). A significantly higher biodiversity in coeliac children's duodenal mucosa was demonstrated by Schippa et al. who also highlighted that the possible pathophysiological role of such microbial differences needs further characterization (35). In addition, many immune phenomena were shown to deteriorate under the effect of changes in the microbiota, as revealed by the correlation among intestinal bacterial overgrowth, increased permeability, and development of non-alcoholic steatohepatitis (36).

Much interest has also been paid to the role of the microbiome in the development of “sterile” inflammation, and recent studies have proved that either depletion of the microbiota or changes in the diet and in the gut microbiome might lead to the improvement of inflammasome-mediated manifestations of autoinflammatory disorders, which are caused by dysregulation of specific components of innate immunity (37). These diseases can be subdivided into monogenic and multifactorial disorders, with the former being caused by mutations of genes involved in the regulation of the innate immune system and the latter by a combination of genetic background and environmental factors (38–40). Given the evidence for the role of the intestinal microbiota in the inflammatory state of IBD, atopic diseases and numerous non-infectious diseases, it has been speculated that intestinal microbial agents might also play a trigger role in the development of other inflammatory disorders which do not have a clearly defined etiology, such as KS.

## Unsolved Problems About the Etiology of Kawasaki Syndrome

The etiology of KS remains obscure, although clinical and epidemiological features suggest a primary infectious cause. Indeed, a self-limited and generally nonrecurring illness that manifests itself by fever, rash, mucositis, conjunctival injection, and cervical adenopathy fits well with an infection. More precisely, clinical features of KS resemble some peculiar infectious diseases, such as streptococcal infections, staphylococcal toxic shock syndrome, and atypical measles (12, 13). Additionally, age distribution, winter-spring seasonality, high rates of KS in siblings, and occurrence of community outbreaks are suggestive of a transmissible childhood disease, though many efforts with conventional bacterial and viral cultures and serological methods as well as animal inoculation studies have failed to identify a final unique infectious agent of KS (41). Reported non-infectious factors associated with KS include carpet shampoo, preexisting eczema, environmental pollution, and house dust mites (42). The higher incidence of KS in Asian populations, presence of familial clustering, and elevated risk of recurrence vs. the risk of a first episode in KS-naïve children are all strong evidence of a genetic contribution to KS susceptibility, probably in association with an infectious trigger (43, 44). However, KS does not appear easily transmittable and does not respond to any antibiotics: these characteristics should contradict a primitively infectious pathogenesis of the illness.

The most current theory about the pathogenesis of KS is that the disease results from an exaggerated immune response toward environmental stimuli occurring in a genetically susceptible child (13). The prominent role played by the immune system in KS is confirmed by the high number of studies revealing the activation of neutrophils and multiple immune cells with overproduction of pro-inflammatory cytokines, including tumor necrosis factor (TNF)-α (45, 46). Manlhiot et al. have very recently proposed a new pathogenetic model of KS in which the disease risk is determined by concurrent interacting processes: genetic susceptibility, habitual exposure to allergens, atmospheric biological particles, and infectious agents (47). A study exploring the immune responses during the acute phase of KS showed increased levels of lipopolysaccharide (LPS) bound to the surfaces of circulating neutrophils via CD14 receptor and found markedly increased levels of the soluble CD14 in the plasma (48). Furthermore, Chen et al. (49) demonstrated that NLRP3 inflammasome activation is associated with the development of coronary arteritis in a mouse model of KS, and that infusion of visfatin, a major injurious adipokine, can activate NLRP3 inflammasome and increase interleukin (IL)-1 production, leading to enhanced endothelial dysfunction. These novel molecular mechanisms of vasculitis mediated by inflammasome activation open more specifically the road to innate immunity pathways in the interpretation of KS (49, 50).

## The Complex Relationship of Kawasaki Syndrome and Infections

Unchallengeable proof that an infection is the starting point of KS is not available. Different epidemiologic studies have supported this hypothesis, based on documented infections by various microorganisms in many cases of KS. Further clues are the occurrence of KS in epidemics, higher incidence during spring and winter and early age at which the disease might be acquired, that is 6 months to 5 years (5). Striking perturbations of many immune pathways occur during the acute phase of KS, which determines a multi-cytokine cascade in the vascular endothelium with focal disruption of small- and medium-sized vessels. However, the exact key steps leading to coronary arteritis are still far from being clarified, though endothelial cell activation; prolonged start-up of neutrophils, CD68^+^ monocyte/macrophages and CD8^+^ lymphocytes; production of oxygen intermediates and lysosomal enzymes; and oligoclonal IgA response by plasma cells all appear to be involved (45, 46, 51–53).

The contribution of viruses to KS has been suggested by ultrastructural studies which found cytoplasmic inclusion bodies containing RNA of viral origin in the bronchial epithelia (54) as well as by studies revealing the detection of intracytoplasmic inclusion bodies containing viral proteins and nucleic acid aggregates (55, 56). Recent investigations have supported the hypothesis that immune responses in KS are oligoclonal rather than polyclonal (as found typically in superantigen-driven responses), and that IgA plasma cells play a crucial role. Indeed, higher levels of IgA have been found in the vasculature of patients with previous KS, indicating an antigen-driven response against an etiologic agent which might have a respiratory or gastrointestinal portal of entry (57).

About half of all KS patients might have one or more respiratory viruses detected by polymerase chain reaction, without any particular predominance, but a positive respiratory viral test or presence of respiratory symptoms at the time of presentation should not be used to exclude the diagnosis of KS (58). Other studies have investigated a potential relationship of KS with coronaviruses, though without finding definite proof of cause and effect (59, 60). Many viruses have been suggested to be implicated in the pathogenesis of KS, such as adenovirus, parvovirus B19, rotavirus, H1N1 influenza virus, Epstein-Barr virus, herpesvirus 6, coxsackie B3 virus, parainfluenza virus type 3, measles virus, dengue virus, human immunodeficiency virus, and varicella-zoster virus, but no significant differences emerged from case-controlled studies (61–69). One of the most difficult infections that are harder to distinguish from KS is caused by adenovirus, due to its frequent incidental detection in KS patients and frequent laboratory finding of increased inflammatory markers (70). In particular, adenovirus has been detected in 8.8 and 25% of cases with complete and incomplete KS, respectively, by Jaggi et al. (71). There are reports that have emphasized the possible relationship of cytomegalovirus (in one child from Turkey) and human bocavirus 1 (in a French cohort of 32 patients) with KS through serologic tests and molecular techniques, but this link might be only casual and misleading (72, 73).

Regarding the bacterial origin of KS, it is debated if the infectious agents might be conventional bacteria or bacteria with superantigen activity. Superantigens (SAgs) are the most powerful T cell mitogens ever discovered, with potent immunostimulatory actions that simultaneously activate the major histocompatibility complex class II molecules and T cell receptors, leading to massive activation of various immune cells. Many studies have investigated the involvement of SAgs in KS. One of the first studies showed the selective expansion of T cell receptor (TCR) Vβ2-bearing T cells in the peripheral blood of children during the acute phase of KS (74). In addition, Leung et al. isolated Staphylococci and Streptococci that produced SAgs (toxic shock syndrome toxin-1 or TSST-1, SST-1, SEB, SEC, SPEB, and SPEC) from the throat, rectum and groin in 25 of 45 patients with untreated KS (56%) in comparison with 13 of 37 control patients (35%) (75).

Matsubara et al. conducted a case-control study with a serological approach based on enzyme-linked immunosorbent assay and measured serum antibodies against staphylococcal enterotoxins, including TSST-1, and streptococcal pyrogenic exotoxin (SPE), such as SPE-A. They showed that KS patients had significant elevation of IgM antibodies against one or more of five SAgs throughout the first to the fourth disease week (76). In a further paper the same authors analyzed the studies regarding the role of SAgs produced by *Staphylococcus aureus* and *Streptococcus pyogenes* in the pathogenesis of KS, finding numerous SAgs implicated, which brought the total number of the known staphylococcal SAgs to over 20 and streptococcal SAgs to 12 (77).

The most recent work that analyzed the relationship between SAgs and KS examined different SAg derived from *Streptococcus pyogenes* in the stools of patients with KS. Stool specimens were obtained from 36 patients with KS during the acute phase and 26 age-matched healthy children. The authors examined genes related to five Sags—SPE-A, SPE-C, SPE-G, SPE-J, and TSST-1—using polymerase chain reaction; throat and stool cultures were assessed to evaluate the presence of *Streptococcus pyogenes* and *Staphylococcus aureus* in KS patients. They reported that at least one of the SAg-related genes was detected more frequently in the stools of children with KS (78). Furthermore, among 358 patients with KS, 54 developed concurrent pneumonia and 12 of these (22.2%) had high titers of anti-*Mycoplasma pneumoniae* antibody (>1:640), suggesting a potential role of *Mycoplasma pneumoniae* in KS and the importance of anti-*Mycoplasma* treatment (79).

Another study has reported a possible role of fungal infections in the development of KS in Japan, San Diego, and the island of Hawaii through an active role of tropospheric wind patterns leaving from Central Asia, in which toxins of *Candida* species are aerosolized (80). In addition, due to the observation that *Candida albicans*-derived substances, such as *Candida albicans* water-soluble fraction (CAWS), induce a coronary arteritis in mice similar to that observed in KS, Sato et al. evaluated the role of a free-β-glucan diet on CAWS*-*induced vasculitis and found that quality of diet might affect the progression of systemic vasculitis (81). CAWS should act as a pathogen-associated molecular pattern in mice and activate lectin pathway of complement by binding to mannose-binding lectin and inducing an acute inflammatory reaction in the vascular system (82, 83).

## What is Known About Microbiota and Kawasaki Syndrome

There are three main reasons that led us to postulate a role of the microbiota in KS. The first is the unsatisfactory microbiological data that limited the association between infections and KS and a lack of evidence of any clear relationship between one or more pathogens with this illness. Second, the most frequent bacteria or viruses associated with KS have a higher prevalence in the overall pediatric population, but only a limited number of children will develop the disease. Third, the association of genetic predisposition and environmental factors in the pathogenetic process of KS makes KS itself similar to other multifactorial diseases.

Currently, the majority of data has found that the composition of the gut microbiota in KS patients differs from healthy subjects. Lee et al. have postulated that the immune system should lose tolerance toward a part of the resident intestinal flora and that environmental factors, i.e., a Western lifestyle or improved public hygiene systems, could transform the commensal flora into a pathogen one, as observed in different gastrointestinal disorders (84).

A recent study about the resident flora and KS was focused on throat flora. Horita et al. (85) assumed that throat flora could be a reservoir of microorganisms triggering KS and, following this hypothesis, they investigated throat microorganisms of KS patients for their content and individual SAg activity. In particular, they collected throat swabs at the start of IVIG infusion in 21 KS patients and compared them with non-KS controls (displaying other febrile illnesses). The results showed no difference in the throat flora between KS and febrile controls even in the mean mitogenic activity of bacteria isolated (85).

The gastrointestinal tract could be one of the primary sites of entry of bacterial toxins in children with KS, and a perturbation in the intestinal microbiota has been linked to the disease's pathophysiology in another study by Yamashiro et al. (86) who investigated the microflora of the small intestine in 15 Japanese patients with KS. The range of bacterial species isolated from jejunal biopsies was characterized by a wider variety of Gram-positive cocci in the acute phase of KS. Notably, 5 kinds of streptococci (*Streptococcus salivarius, Streptococcus mitis, Streptococcus oralis, Streptococcus sangius*, and *Gemella haemolysans*) and 2 kinds of staphylococci (*Staphylococcus capitis* and *Staphylococcus hyicus*) were isolated only from KS patients, suggesting that some antigens inducing a delayed-type hypersensitivity reaction in the mucosa might inundate the body by breaching the barrier of the small intestinal mucosa of KS patients (86). In fact, Nagata et al. (87) investigated cell surface phenotypes of mononuclear cells and enterocytes in the jejunal mucosa of 16 Japanese patients with KS and in 10 patients with diarrhea due to cow's milk protein intolerance. Both HLA-DR+CD3+ and DR+CD4+ cells were significantly increased, and CD8+ cells significantly reduced in the lamina propria of KS patients during the acute phase compared with patients with cow's milk protein intolerance. These cell patterns returned to normal in the convalescent phase of KS. The authors concluded that a delayed-type hypersensitivity reaction was indeed present in the small intestinal mucosa of KS patients (87).

Due to the fact that the gastrointestinal tract is the largest interface between microbial factors and their host, containing the largest proportion of bacteria and the largest amount of lymphoid tissue in the body, it was hypothesized by Eladawy et al. that the intestinal milieu could be altered in children with KS, and indeed KS patients have a higher incidence of gastrointestinal symptoms and complications (88). Specifically, Takeshita et al. (89) evaluated 20 patients with KS, 20 patients with acute febrile diseases and 20 healthy children, finding that the incidence of *Lactobacilli* isolated from KS patients (2/20, 10%) was significantly lower (*p* < 0.001) than in the other cohorts. Moreover, no significant differences in the presence of *Staphylococcus, Streptococcus, Enterococcus, Enterobacteriaceae, Bifidobacterium, Clostridium, Veillonella*, or *Bacteroides* were found among the three groups. Conversely, the presence of *Eubacterium* and *Peptostreptococcus* was significantly higher in KS patients than in patients with other febrile diseases (*p* < 0.01 and *p* < 0.05, respectively), though no significant differences were observed between KS patients and healthy children (89). This observation confirmed that the majority of *Lactobacillus* species are anti-inflammatory and beneficial for health, particularly during the first years of life, due to their action in protecting against colitis, reducing pro-inflammatory cytokines such as TNF-α, IL-1, or IL-6, and increasing the subsets of regulatory T cells (90). In fact, several studies have largely described the specific action of *Lactobacilli* in maintaining the epithelial homeostasis of the gut and their striking anti-inflammatory potential (91, 92).

Nagata et al. (93) also studied the role of the gut microbiota in KS pathogenesis via SAgs and heat shock proteins (Hsps) produced by gut bacteria: the authors evaluated Sags and Hsps released by microorganisms isolated from the jejunal mucosa of 19 children with KS compared with 15 age-matched healthy controls, identifying 13 strains of Gram-negative microbes from patients with KS, which produced a large amount of Hsp60, having the power to induce an over-secretion of pro-inflammatory cytokines. They also identified 18 strains of Gram-positive cocci with SAg properties which induced the expansion of Vβ2 T cells *in vitro* (93). This microbiological analysis disclosed different causative bacteria with a final common pathway of immune activation, which might contribute to the final development of KS.

In order to evaluate the differential microbiota composition of KS patients, Kinumaki et al. (94) performed a metagenomic analysis using non-culture-based methods on feces. Their study included 28 KS patients (15 males and 13 females, aged 1–114 months, with a median age of 25 months); the time of admission to the study was defined as the acute phase, while 4–6 months after the onset of KS was considered as the non-acute phase. The authors collected a total of 56 samples−28 samples each for both acute and non-acute phases. It was demonstrated that the genera *Ruminococcus, Roseburia*, and *Faecalibacterium* were mostly predominant during the non-acute phase, while a higher presence of *Streptococcus* spp., including *Streptococcus pneumoniae, pseudopneumoniae, mitis, oralis, gordonii*, and *sanguinis*, was detected in the fecal samples during the acute phase (94).

This novel interpretation of a disease can be shared by other conditions, as liver cirrhosis and Sjögren syndrome, in which major changes of gut microbiota with higher proportions of *Streptococcus* spp. have been demonstrated (95, 96). These findings taken as a whole suggest that many other immune-mediated disorders are likely to be connected to an abnormal bacterial colonization of the intestinal tract, with a main role for *Streptococcus* spp., and that changes in the gut microflora composition might promote systemic and extra-intestinal inflammation. Therefore, all presented studies confirm the concept that an imbalance in the gut microbiota might directly or indirectly interfere with the normal functions of the immune system, and that the interaction with other environmental factors, mainly infectious agents, might lead to the final development of KS.

Table 1 shows the characteristics of the microbiota in children with KS, as emerging by the most relevant studies dedicated to this issue.

**Table 1:** Perturbation in the intestinal microbiota of patients with Kawasaki syndrome.

**References**	**Methods**	**Site**	**No of patients**	**Results**
Horita et al. (85)	Culture	Throat	21 patients with KS, 20 with other febrile illnesses	No difference
Takeshita et al. (89)	Culture	Gut	20 patients with KS, 20 patients with acute febrile diseases, 20 healthy children	↓*Lactobacillus*, ↑*Eubacterium^a^*, ↑*Peptostreptococcus^a^*
Nagata et al. (93)	Culture	Gut	19 patients with KS, 15 patients with food-sensitive enteropathy in remission	↑ Gram-negative producing hsp60, ↑ Gram-positive cocci with superantigenic properties
Kinumaki et al. (94)	Metagenomic analysis on feces	Gut	28 KS patients, 28 samples during acute-phase, 28 samples in non-acute phase	↑*Ruminococcus*^b^, ↑*Roseburia*^b^, ↑*Faecalibacterium*^b^, ↑*Streptococcus* spp^c^

a *Higher in KS patients than in other febrile illness (no differences were observed between KS patients and healthy children)*.

b *Higher in KS patients during non-acute phase of the disease*.

c *Higher in KS patients during acute phase of the disease*.

## Conclusive Remarks and Future Perspectives

As no etiologic agent has ever been accused of being directly involved in the etiology of KS, basic research evaluating the pathogenic mechanisms of this disease will probably provide better therapies and probably consent to identify the most vulnerable hosts and protect them. A potential relationship between eubiosis and the protean functions of immunity has been contemplated by different studies, and conversely a relationship should exist between dysbiosis and immunity dysfunction shown by various diseases (97). It is strengthened that heterogeneity and abnormalities in the intestinal microflora composition may trigger or contribute to the development of specific diseases, and an increasing amount of research and microbiologic observations have led to a role of the intestinal microbiota for KS to be postulated. While we are becoming convinced that a role of the microbiota is likely, we do not know exactly the basic mechanism of how the microbiota should act. The principal obstacle of today's medical literature is to understand if the microbiota modification during KS can cause the disease or if it is a mark and a consequence of the disease itself, and if modulating the microbiota/microbiome might represent a future target of therapy in KS. This raises the hypothesis of targeting intestinal microflora in order to restore eubiosis through the rational use of antibiotics, xenobiotics, probiotics and nutrients. While multicenter trials and registries may allow us to improve the general outcome of KS, further in-depth analysis in larger cohorts of affected children will probably unravel the tangled pathogenesis of this mysterious disorder and find new targets for identifying disease-modifying agents or more specific therapies.

## Author Contributions

SE, IP, and DR contributed to all stages of the preparation of this manuscript, including conception and writing. All authors approved the final submitted version of the manuscript.

### Conflict of Interest Statement

The authors declare that the research was conducted in the absence of any commercial or financial relationships that could be construed as a potential conflict of interest.
